# Effects of Culture and Gender on Judgments of Intent and Responsibility

**DOI:** 10.1371/journal.pone.0154467

**Published:** 2016-04-28

**Authors:** Jason E. Plaks, Jennifer L. Fortune, Lindie H. Liang, Jeffrey S. Robinson

**Affiliations:** 1 Department of Psychology, University of Toronto, Toronto, Ontario, Canada; 2 School of Liberal Arts, Humber College, Toronto, Ontario, Canada; 3 Department of Industrial/Organizational Psychology, University of Waterloo, Waterloo, Ontario, Canada; University of L'Aquila, ITALY

## Abstract

Do different cultures hold different views of intentionality? In four studies, participants read scenarios in which the actor’s *distal intent* (a focus on a broader goal) and *proximal intent* (a focus on the mechanics of the act) were manipulated. In Studies 1–2, when distal intent was more prominent in the actor’s mind, North Americans rated the actor more responsible than did Chinese and South Asian participants. When proximal intent was more prominent, Chinese and South Asian participants, if anything, rated the actor more responsible. In Studies 3–4, when distal intent was more prominent, male Americans rated the actor more responsible than did female Americans. When proximal intent was more prominent, females rated the actor more responsible. The authors discuss these findings in relation to the literatures on moral reasoning and cultural psychology.

## Introduction

Intentionality features prominently in philosophers’ and laypeople’s calculations of moral responsibility, blame, and punishment [1–3]. In general, people judge an actor who caused an outcome intentionally to be more responsible than an actor who caused the identical outcome unintentionally (e.g., [4–7]). Moreover, virtually all legal systems distinguish between, for example, murder versus manslaughter.

Relatively little research, however, has investigated how laypeople determine intentionality to begin with (cf. [8]). One exception is a model proposed by Malle and Knobe [3], which identified “awareness of the act while the person is performing it” as one of five components of the folk theory of intentionality. The present research builds on this idea by focusing on different ways of construing the actor’s awareness.

Researchers have identified two central ingredients in laypeople’s mental models of intentional action: *proximal intent* and *distal intent* [9–10]. Proximal intent is present in the actor’s mind to the extent that the actor performs the act with her mind focused on the mechanics of the action, or the means (e.g., pulling the trigger). Distal intent is present in the actor’s mind to the extent that she performs the act with her mind focused on the broader aim, or the end (killing John).

In the present studies, we examine cultural variation in the weight perceivers place on proximal and distal intent when judging an actor’s degree of responsibility. We test the hypothesis that North Americans place more weight on whether the actor was thinking primarily about the larger aim (i.e., distal intent). At the same time, we hypothesized that East Asians and South Asians would place more weight on whether the physical act was executed with awareness and control (i.e., proximal intent). Before describing the rationale for these hypotheses, we turn first to a fuller description of the general framework.

### The Proximal Intent / Distal Intent Framework

To begin to illustrate the components of an intentional act, consider the following scenario:

Alex wants to kill his ex-girlfriend Linda. He formulates a plan to drown her in the lake during a camping trip they attend with the same group of friends every year. Knowing she can swim, Alex plans to tie her up with a rope before pushing her into the water. Alex convinces Linda to go for a boat ride with him. As they are about to get into the boat, docked in a secluded area by the lake, Alex takes a rope from the boat and begins to tie her up. Linda manages to free herself and push Alex into the water. Then, while running away, Linda trips, hits her head on the dock, falls into the water unconscious, and drowns.

In this scenario, although Alex desired Linda’s death, planned Linda’s death, and clearly played a role in causing the death, the deathblow occurred in an unplanned and uncontrolled manner. Pizarro, Uhlmann, and Bloom [7] found that participants rated actors in such “causally deviant” scenarios less responsible than actors in scenarios in which the same outcome occurred according to plan. Such data suggest that laypeople’s representations of intentionality and responsibility are not necessarily binary (“intentional” vs. “unintentional”). Instead, they appear to be sensitive to incremental gradations of responsibility.

According to the Proximal Intent / Distal Intent (PIDI) framework [9–10], laypeople perceive moral responsibility in a graded fashion because their model of intentionality contains two key subcomponents: proximal intent and distal intent. Proximal intent refers to exercising awareness and control over the application of force (i.e., doing it “on purpose”). That is, the actor’s mind is focused on the successful physical execution of the act. Distal intent refers to performing the act as a means to an end. That is, during the act, the actor’s mind is focused on a larger aim, beyond the physical movement. In other words, proximal intent describes intentionality regarding the means, whereas distal intent describes intentionality regarding the end.

Philosophers and legal scholars have made similar distinctions between, for example, “oblique” versus “direct” intention [11], “intention” versus “intention-in-action” [12], “prospective” versus “concurrent” intention [13], and “bare intention” versus “intentional action” [14]. As valuable as these contributions have been, there have been, to our knowledge, few attempts to operationalize and measure folk versions of these concepts using contemporary social psychological methods.

### PI and DI May Vary Independently

**Fig 1:**
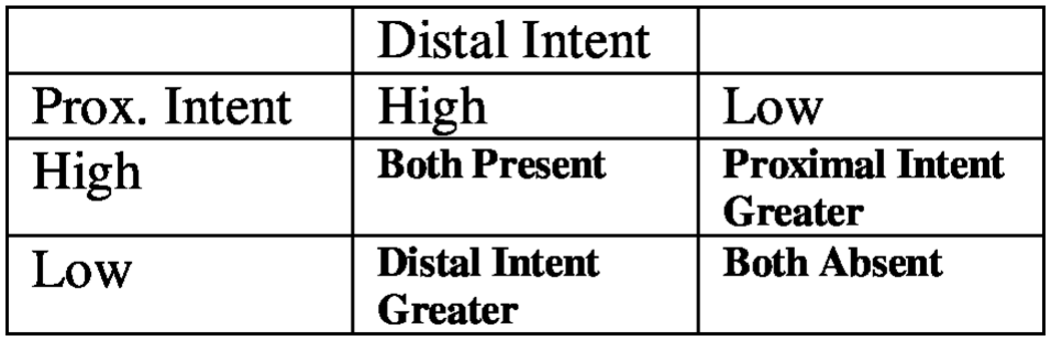
A representation of proximal and distal intent as independent dimensions.

For most mundane intentional acts, both proximal and distal intent are simultaneously present in the actor’s mind (e.g., Wishing to eat a cookie, Joe lifts a cookie into his mouth with awareness and control.). However, a central assumption of the PIDI framework is that proximal and distal intent may vary independently. Thus, at the moment of the act, it is possible for the actor to have both forms of intent equally present in conscious awareness, or one form of intent represented more prominently than the other. For example, when the actor’s mind is focused primarily on a larger goal, but the outcome is reached by accident, the act can be said to have occurred with distal intent more prominent than proximal intent. (The Alex-Linda scenario above is one example.) On the other hand, an act that occurs with the actor focused primarily on the means of execution, and less on the broader aim, has occurred with proximal intent more prominent than distal intent. For example, imagine that Alex, who wants to kill Linda, goes to a gun range for target practice. While focusing intently on his shooting technique, the bullet shoots out of the gun and hits Linda. Thus, although Alex may possess a longstanding *desire* to kill Linda, at the moment of this particular act, Alex’s distal intent is to ‘hit the target’, not to ‘kill Linda’. In summary, it is possible to represent the presence or absence of proximal intent and distal intent using a 2 (distal intent: present vs. absent) X 2 (proximal intent: present vs. absent) framework. (Fig 1)

### Caveats

Although in the present studies we operationalized proximal and distal intent dichotomously (high vs. low) for the sake of simplicity, people most likely represent the presence/absence of proximal and distal intent in a continuous manner. In other words, the experimental conditions in the present studies refer to relative, not absolute differences. Thus, at moments when one form of intent is more prominent in the actor’s mind, the other form of intent is never entirely absent.

In addition, we do not claim that proximal and distal intent are the sum total of ingredients of an intentional act. For example, research has illustrated the importance of the actor’s *belief* [15], *desire* [16], *skill* [17], and ability to *foresee the consequences* of the act [18, 12]. These elements clearly play important roles. Yet each refers to mental states or conditions that precede the focal act. Less research has focused on the actor’s intent-related thoughts during the actual execution of the act. Consider, for example, the distinction between distal intent and “desire”. Desire refers to a preferred or wished-for outcome. People may, however, harbor a particular desire without acting on it (e.g., [19]). Distal intent, in contrast, is “desire” put into action; it is the mental representation of the end that a physical movement is addressing. Thus, distal intent, by definition, can only be present during an act. Similarly, distal intent differs from the legal and philosophical concepts “motive” and “plan” [18, 20]. Whereas motive and plan refer to pre-existing thoughts and conditions that are independent of the act itself, distal intent can only be present when the act is occurring.

### Initial Evidence for the PIDI Framework

Plaks and colleagues [9–10] presented participants with scenarios that featured the independent manipulation of the actor’s proximal and distal intent. In all seven studies (total N > 1,500), participants assigned the most responsibility to the actor with both forms of intent in mind, a moderate amount of responsibility to the actor with one form of intent more prominent than the other, and the least responsibility to the actor with neither form of intent in mind.

In addition, the researchers identified social psychological variables that predicted whether perceivers’ judgments would be more influenced by the presence or absence of proximal intent or the presence or absence of distal intent. One such variable was psychological distance. When the act occurred in a distant location or the distant past, participants were more focused on distal intent (i.e. whether the actor had the malevolent goal in mind) than proximal intent (i.e. whether the act was performed on purpose or by accident). When the act occurred in a near location or in the recent past, participants were more focused on whether the act was performed on purpose or by accident than whether the actor had the malevolent goal in mind at the critical moment [9–10].

### Culture and Perceptions of Action and Agency

The present studies extend this line of research by examining whether culture-related variables are associated with greater sensitivity to either proximal intent or distal intent. We turn our attention to culture for two reasons. First, the use of culture as an independent variable may help to isolate specific assumptions about human agency that lead people to place more weight on either proximal intent or distal intent. Second, a demonstration of meaningful cultural differences in intent perception would be noteworthy in its own right and may inform the field’s understanding of how culture shapes moral judgment.

Why might one expect cultural differences in the perception of intent? In several papers, Savani, Markus, and colleagues provided evidence that Americans—as a consequence of their “disjoint” model of agency [21]–tend to view actions as emanating from the individual’s idiosyncratic “preferences, beliefs, and goals” [22]. In contrast, South Asians—as a consequence of their “conjoint” model of agency—are more likely to view actions as “responsive to the situation, to social roles, and to expectations of other individuals” ([22]; see also [23]).

In other words, North Americans are more likely than South Asians to view a given action as a *choice* [24]. Viewing an action as an individual choice assumes that the actor is striving for a particular outcome. After all, people rarely initiate an action with no outcome in mind. Indeed, an act is perceived to be more ‘act-like’ if it is preceded by the choice to pursue a specific outcome [25].

Put differently, viewing an action as a choice suggests viewing it as a means to an end. This perspective should highlight the actor’s distal intent. Thus, if North Americans (relative to South and East Asians) show a greater tendency to view actions as choices, they should place greater emphasis on the actor’s distal intent. As such, Hypothesis #1 was that North Americans’ assessments of responsibility would be more sensitive to the presence or absence of distal intent in the actor’s mind. In contrast, to the extent that South and East Asians are more inclined than North Americans to view actions as responses to situational or normative pressures, the actor’s idiosyncratic wishes hold less relevance. With distal intent minimized, South and East Asians’ judgments of responsibility should be based more heavily on other components of intentionality—including the degree to which the actor executed the movement with awareness and control (i.e. proximal intent). Thus, Hypothesis #2 was that South and East Asians’ assessments of responsibility would be more sensitive to the presence or absence of proximal intent. [Note that this hypothesis differs from the well-known effect of culture on ‘person’ versus ‘situation’ attributions (e.g., [26–27] in that both forms of intent, which occur inside the actor’s mind, would likely be categorized as elements of the ‘person’.]

Indirect evidence for the hypotheses comes from studies on religion and the perception of intent (e.g., [5, 19]). For example, Laurin and Plaks [5] compared adherents of orthopraxic (action-focused) religions (e.g., Hinduism) to adherents of orthodox (faith-focused) religions (e.g., Protestantism). The authors found that when an actor committed a homicide intentionally, Hindus and Protestants did not differ in their punishment of the actor. However, when the actor caused the same outcome unintentionally, Hindus punished the actor significantly more than did Protestants. To the extent that orthopraxic religiosity is more prevalent in collectivist cultures and orthodox religiosity is more prevalent in individualistic cultures (e.g., [19]), these results may suggest a link between, on the one hand, collectivism and a focus on actions and outcomes and, on the other hand, individualism and a focus on the actor’s thoughts, wishes, and plans.

### Gender and Perceptions of Agency

In Studies 3 and 4, we turned to gender as the predictor variable for two reasons. First, although gender clearly differs from culture in any number of important ways, there have been several documented associations between the two [28–29]. For example, both Western culture and men have been reliably associated with a similar constellation of constructs: individualism, independence, autonomy, and agency. On the other hand, Eastern cultures and women have been associated with collectivism, interdependence, communalism, and relationalism [30–32]. Although researchers have helpfully noted that males and females display different forms of interdependence (“collective” versus “relational”; [29]), the preponderance of evidence points to an overall male-independence / female-interdependence link. Thus, the use of gender as an independent variable may represent an alternative way to test our cultural hypotheses. Specifically, we expected that, within a U.S. sample, males would be more sensitive than females to the presence or absence of distal intent, whereas females would be more sensitive than males the presence or absence of proximal intent. Such a pattern would support the notion that it is culture *per se* (as opposed to nationality or ethnicity) that is behind the effect.

## Study 1

In Study 1, we tested our hypotheses by comparing undergraduate students in China and Canada.

Note: All studies reported here were reviewed and approved by the University of Toronto Office of Research Ethics Board prior to being conducted. All participants gave consent to participate either by signature (if participating in person) or by ticking a box (if participating online). The text of the consent statements was identical in both cases. Consent data were stored separately from study data. The University of Toronto Research Ethics Board approved this consent procedure.

### Participants

A total of three hundred and fifty-nine participants (142 females, 215 males, 2 unspecified) participated. Canadian participants (156 undergraduates at the University of Toronto, mean age = 20.5) were Introductory Psychology students who received extra course credit. They were recruited through a voluntary experiment participation website maintained by the Department of Psychology. Chinese participants (203 undergraduates at the University of Jilin, in the northern city of Changchun, mean age = 20.6) were recruited verbally in a single lecture setting. See Table 1 for additional demographic data for each sample. The Canadian sample size was determined by calculating the amount of participants needed to reach at least 80% power based on effect sizes reported in previous, similar studies (e.g., [10]). The Chinese sample size was determined by including all of the students present in one lecture. No participants in either sample were excluded. To create the Chinese stimuli, all materials were translated from English to Chinese by one expert translator and then back-translated to English by a second expert translator. This process revealed no translation discrepancies. (In the Chinese versions, the characters were given Chinese names.)

**Table 1:** Demographic data in all studies.

**Study 1**	Chinese (*M* age = 20.6)	Canadian (*M* age = 20.5)
	N (%)	N (%)
Female	45 (22)	97 (63)
Male	157 (78)	58 (37)
African	Information	3 (2)
Caucasian	Not	65 (41)
East Asian	Available	42 (27)
Hispanic		1 (1)
Middle Eastern		5 (3)
South Asian		37 (20)
other or not reported		4 (2)
**Study 2**	South Asian (M age = 28.1)	N. American (M age = 35.2)
	N (%)	N (%)
Female	136 (61)	34 (49)
Male	86 (39)	35 (51)
African	Information	1 (2)
Caucasian	Not	52 (75)
East Asian	Available	7 (10)
Hispanic		1 (1)
Middle Eastern		2 (3)
South Asian		8 (10)
other or not reported		1 (1)
H. Collectivism (mean)	7.01	6.09
V. Collectivism (mean)	6.33	5.46
H. Individualism	6.65	7.15
V. Individualism	6.18	5.92
Religiosity (mean)	6.18	3.55
**Study 3**	Female (M age = 38.57)	Male (M age = 35.72)
	N (%)	N (%)
	198 (51)	190 (49)
African	18 (8)	12 (6)
Caucasian	129 (65)	138 (72)
East Asian	9 (5)	7 (4)
Hispanic	7 (4)	10 (5)
Middle Eastern	1 (0)	1 (0)
South Asian	3 (1)	5 (3)
other or not reported	31 (15)	18 (9)
Religiosity (mean)	4.16	3.69
**Study 4**	Female (*M* age = 36.47)	Male (*M* age = 35.72)
	N (%)	N (%)
	262 (55)	206 (45)
African	24 (9)	18 (9)
Caucasian	174 (68)	143 (69)
East Asian	6 (2)	7 (3)
Hispanic	12 (5)	12 (6)
Middle Eastern	3 (1)	2 (1)
South Asian	8 (3)	6 (3)
other or not reported	27 (11)	19 (9)
Religiosity (mean)	3.56	3.15

### Procedure

Participants were randomly assigned to read one of four scenarios (modified from [7]). In the Both Present condition, an actor (“Barbara”) had the goal of killing her husband (“John”) in mind as she successfully executed her plan to poison him during dinner at a restaurant. In the Distal Intent Greater condition, Barbara had the goal (killing John) prominently in mind at the moment when the outcome occurred in a causally deviant manner (John died not because of the poison but because of an allergy to a second dish). In the Proximal Intent Greater condition, the goal of killing John was not prominent in Barbara’s mind at the moment when she purposefully performed the action (pouring the poison) that killed John. In the Both Absent condition, both forms of intent were not prominent in Barbara’s mind: she was not at that moment thinking about killing John and she did not perform the deathblow on purpose. In other words, the proximal intent manipulation was whether the deathblow was performed under Barbara’s control versus outside of Barbara’s control. The distal intent manipulation was whether Barbara’s thoughts at the critical moment were on killing John versus a different topic. Care was taken to ensure that the scenarios were free of potential flaws identified by Pizarro et al. [7] (e.g., the actor’s intention contributed to causing the outcome, the actions were not subject to last minute changes-of-mind). For the complete scenarios, see S1 Appendix.

#### Dependent Measures

After reading one of the four scenarios (randomized), participants indicated their rating of the actor on the following items (0–5 scales): 1. How much moral responsibility does Barbara deserve for what happened to John? 2. How intentional was Barbara’s action? 3. How negatively should Barbara be judged? 4. How much blame goes to Barbara for what happened? These items have been used in previous studies (e.g., [9–10]) and were included to assess whether participants’ judgments would distinguish between intentionality versus general negativity and blame toward the actor (e.g., [33]).

### Results

#### Overall Effects (Collapsed Across Culture)

Responses to the four moral judgment items questions were significantly intercorrelated (Cronbach’s α = .84). Thus they were aggregated into a moral judgment index. Scores on the index were submitted to a 2 (culture: Canadian vs. Chinese) x 2 (distal intent: high vs. low) x 2 (proximal intent: high vs. low) between-subjects ANOVA. Replicating previous work [9–10], the ANOVA revealed significant main effects for distal intent, *F*(1, 359) = 27.77, *p* < .001, η_p_^2^ = .07, and proximal intent, *F*(1, 359) = 25.14, *p* < .001, η_p_^2^ = .07.

Simple effects comparisons revealed that participants rated the actor with both forms of intent present more responsible (*M* = 4.38) than the actor with both forms of intent absent (*M* = 2.94), *F*(1, 359) = 60.38, *p* < .001, η_p_^2^ = .20. In addition, participants rated the actor with distal intent more prominently in mind than proximal intent (*M* = 3.98) less responsible than the actor with both present, *F*(1, 359) = 4.11, *p* < .05, η_p_^2^ = .03, and more responsible than the actor with both absent, *F*(1, 359) = 30.83, *p* < .001, η_p_^2^ = .12. Similarly, participants rated the actor with proximal intent more prominently in mind than distal intent (*M* = 3.98) less responsible than the actor with both present, *F*(1, 359) = 4.38, *p* < .05, η_p_^2^ = .03, and significantly more responsible than the actor with both absent, *F*(1, 359) = 32.22, *p* < .001, η_p_^2^ = .13. Thus, in the aggregate, judgments of responsibility varied according to the type of intent in the actor’s mind. When both forms of intent were prominent in the actor’s mind, participants rated her most responsible, when one form of intent was more prominent than the other, participants rated her moderately responsible, and when both forms of intent were largely absent from her mind, participants rated her least responsible.

#### Effects Involving Culture

Did participants’ cultural identity (Canadian vs. Chinese) influence this overall pattern? First, the omnibus analysis revealed an overall main effect of culture, *F*(1, 359) = 17.67, *p* < .001, η_p_^2^ = .50 indicating that Canadians generally judged Barbara more harshly. However, a significant proximal intent x culture interaction, *F*(1, 359) = 12.17, *p* < .001, η_p_^2^ = .03, and a significant culture x proximal intent x distal intent interaction, *F*(1, 359) = 9.03, *p* < .01, η_p_^2^ = .02, indicated that the difference between the Canadian and Chinese participants varied according to the type of intent in the actor’s mind.

To better understand the interactions, we compared the Canadian versus Chinese participants’ ratings within each of the four scenario conditions. As depicted in Table 2, Canadians judged the target more harshly than did Chinese participants when both forms of intent were present, *F*(1, 359) = 6.11, *p* < .05, η_p_^2^ = .07, when both forms of intent were absent, *F*(1, 359) = 23.68, *p* < .001, η_p_^2^ = .13, and when distal intent was greater than proximal intent, *F*(1, 359) = 8.34, *p* < .01, η_p_^2^ = .12. However, this difference, if anything, reversed when proximal intent was greater than distal intent: Chinese participants (*M* = 4.19) rated the actor marginally more responsible than did Canadian participants (*M* = 3.73), *F*(1, 359) = 3.25, *p* = .07, η_p_^2^ = .06.

**Table 2:** Moral sanction of the actor. (Standard deviations in parentheses.)

	Chinese			Canadian	
	Distal Intent			Distal Intent	
Prox. Intent	High	Low	Prox. Intent	High	Low
High	4.10 (1.48)	4.19 (0.76)	High	4.74 (0.50)	3.73 (1.03)
Low	3.61 (1.16)	2.50 (1.72)	Low	4.38 (0.87)	3.70 (1.20)

In an analysis comparing the Proximal Intent Greater versus Distal Intent Greater conditions, a significant 2 (culture: Chinese vs. Canadian) x 2 (scenario: Proximal Intent Greater vs. Distal Intent Greater) interaction, *F*(1, 172 = 17.34, *p*< .001, η_p_^2^ = .09, confirmed that Chinese and Canadian participants differed in their view of which single type of intent carried more weight. Canadian participants judged the target more harshly in the Distal Intent Greater condition, while Chinese participants, if anything, judged the target more harshly in the Proximal Intent Greater condition.

In sum, the Study 1 data are consistent with the notion that North Americans’ judgments of responsibility are more sensitive to the presence or absence of distal intent while East Asians’ judgments are, if anything, more sensitive to the presence or absence of proximal intent. These effects emerged despite the fact that 27% of the Toronto sample self-identified as ethnically East Asian (primarily Chinese). This suggests that the observed effects are unlikely to be attributable to ethnicity per se.

## Study 2

Study 2 had two aims. First, for the sake of generalizability, we tested whether the pattern would replicate with a new set of scenarios. Second, we tested whether the pattern would extend to a different operationalization of culture (i.e., replacing East Asian with South Asian). A parallel between East Asians’ and South Asians’ responses would suggest that a common element of these cultures—perhaps collectivism—plays an important role in the cross-cultural effect.

### Participants

A total of 309 participants (181 males, 126 females, 2 unspecified; mean age = 30.90) were recruited via an online survey service (Amazon Mechanical Turk) in exchange for a payment of US $0.50. All materials and responses were written in English. (South Asian Mechanical Turk participants tend to possess high proficiency in English.) [Note: The original intention was to collect data from 300 North Americans, but due to experimenter error, the sample was not restricted to North Americans. When researchers do not explicitly set the Mechanical Turk sample to be restricted to North Americans, a significant number of English-speaking South Asians tend to participate. The sample sizes of North Americans and South Asians reported below reflect this outcome. Once the data were collected, we decided to capitalize on the opportunity to further test our cross cultural hypothesis with the data at hand.]

### Procedure

First, participants completed a series of demographic items. Culture was assessed with the following open-ended questions: “What is your cultural background?”, “What is your ethnicity?”, and “What is your nationality?” Based on participants’ responses, as well as the nation associated with each respondent’s IP address, two coders assigned each participant to a single ‘best fit’ cultural group. Discrepancies (n = 3) were resolved through discussion. Participants who either failed to complete any of the culture/ethnicity questions or provided incoherent responses were not included in the analyses. The net result was two primary groups, which we labeled South Asians (n = 224) and North Americans (n = 69). The North Americans hailed from the United States (n = 54) and Canada (n = 15). Fifty-nine (86%) of the North Americans reported being ethnically Caucasian. The South Asians hailed from India (n = 159), Pakistan, (n = 45), and Bangladesh (n = 20). Various local ethnicities (e.g., Gujurati, Tamil, Maharastrian) were typed into a question box labelled “ethnicity”, with no single ethnicity clearly predominating. The mean age of the North Americans (*M* = 35.23) was higher than that of the South Asians (*M* = 28.06), *t*(289) = 5.31, *p* < .01. Participants (n = 16) who provided no information regarding their culture or responded unhelpfully (e.g., “I am human”) were not included in the analyses.

In addition, participants completed the Triandis and Gelfand [32] measure of individual/collectivism. This widely used measure contains separate dimensions for individualism vs. collectivism and “horizontal” vs. “vertical” orientations. The horizontal dimension refers to the degree to which individuals view themselves as independent of/interdependent with others who are equal in status. The vertical dimension refers to the degree to which individuals view themselves as independent of/interdependent with others who differ in status. (For a full description of these dimensions, see [34].) Participants also rated themselves (on a 1–6 scale) on the following item, “How religious are you?”

Next, participants read one of four scenarios. In the Both Present condition, an actor (“Alex”) had the goal of killing his ex-girlfriend (“Linda”) in mind as he purposefully executed his plan to tie her up and drown her in a lake. In the Distal Intent Greater condition, Alex had the goal (killing Linda) in mind at the moment when the outcome occurred in a causally deviant manner (Linda died while attempting to flee). In the Proximal Intent Greater condition, he did not have the goal of killing Linda in mind at the moment when he purposefully performed the action (pulling a rope) that killed Linda. In the Both Absent condition, he had neither the goal in mind, nor performed the deathblow on purpose. In short, distal intent was manipulated by varying whether Alex’s mind at the moment of the deathblow was focused on killing Linda or on a different aim. Proximal Intent was manipulated by varying whether the deathblow occurred under Alex’s control versus not under Alex’s control. All other content was held constant. (Note that even though Alex’s longstanding desire to kill Linda was stated at the beginning of every scenario, in the conditions when Distal Intent was largely absent, the intention to kill Linda was explicitly *not* in his mind.) (See S1 Appendix)

#### Dependent Measures

After reading one of the four scenarios, participants provided ratings (on 0–6 scales) on the same items used in Study 1: 1. How much moral responsibility does Alex deserve for what happened to Linda? 2. How intentional was Alex’s action? 3. How negatively should Alex be judged? 4. How much blame goes to Alex for what happened?

### Results

#### Data Management (Prior to Analyses)

Because reasonable concerns have been raised about the quality of data collected via Mechanical Turk [35–36], we performed a series of operations on the entire data set prior to conducing any statistical analyses. First, we checked for duplicate IP addresses to eliminate participants who participated more than once. Then we performed two attention checks: 1. We searched for participants who provided the same response to every single question. 2. We compared the item requesting the participant’s birth year to the age reported in a previous question. Taken together, these steps resulted in the elimination of 11 participants. Analyses that include these participants yield results that do not differ significantly from the results reported below.

#### Overall Effects (Collapsed Across Culture)

Because the four judgment questions were highly intercorrelated (α = .76), they were averaged to create a moral sanction index. Participants’ scores on the index were submitted to a 2 (culture: North American vs. South Asian) x 2 (distal intent: high vs. low) x 2 (proximal intent: high vs. low) between-subjects ANOVA.

As in Study 1, this analysis revealed significant main effects for distal intent and proximal intent, both *F*s>9.10, both *p*s < .001, both η_p_^2^s >.03. Participants rated the actor with both forms of intent most responsible (*M* = 4.87) and the actor with both absent (*M* = 3.38) least responsible, *F*(1,282) = 35.71, *p* < .001, η_p_^2^ = .22. They rated the actor in the Proximal Intent Greater condition morally responsible to a degree that was between the Both Present and Both Absent actors (both *F*s>5.10, *p*s < .05, η_p_^2^s>.04). Likewise, they rated the actor with Distal Intent Greater morally responsible to a degree that was between the Both Present and Both Absent actors (both *F*s>7.77, *p*s < .01, η_p_^2^s>.06).

#### Effects Involving Culture

A distal intent x culture interaction, *F*(1, 282) = 13.05 *p* < .001, η_p_^2^ = .05, indicated that the two cultural groups were differentially influenced by distal intent. As in Study 1, we probed the interaction by comparing the North Americans and South Asians within each of the four scenarios. When the actor had proximal intent more prominent than distal intent (Proximal Intent Greater), South Asians (*M* = 4.25) rated him more responsible than did North Americans (*M* = 3.52), *F*(1, 282) = 4.38, *p* < .05, η_p_^2^ = .06. However, when the actor had distal intent more prominent than proximal intent (Distal Intent Greater), North Americans (*M* = 4.61) rated him marginally more responsible than did South Asians (*M* = 4.10), *F*(1, 282) = 3.48, *p* = .06, η_p_^2^ = .04. (See Table 3) This pattern was confirmed by a significant 2 (culture: North American vs. South Asian) x 2 (scenario: Proximal Intent Greater vs. Distal Intent Greater) interaction, *F*(1, 282) = 3.44, *p* < .05, η_p_^2^ = .05.

**Table 3:** Moral sanction of the actor. (Standard deviations in parentheses.)

	South Asian			N.American	
	Distal Intent			Distal Intent	
Prox. Intent	High	Low	Prox. Intent	High	Low
High	4.75 (1.09)	4.25 (1.06)	High	5.15 (0.88)	3.52 (0.87)
Low	4.10 (1.05)	3.86 (0.97)	Low	4.61 (1.27)	3.38 (1.37)

Thus, the pattern in Study 2 generally tracked the pattern in Study 1: different cultural backgrounds were associated with differential weighting of distal intent and proximal intent. North Americans focused more on whether the actor’s malevolent goal was in mind at the moment of the death, whereas South Asians focused more on whether the action itself was performed with awareness and control.

#### Mediational Role of Collectivism

[Note: We initially conducted separate analyses for vertical and horizontal forms of collectivism and individualism. The two forms of collectivism yielded statistically equivalent patterns. Thus we report the collectivism data collapsed across the vertical and horizontal dimensions. The two dimensions of individualism did diverge and are described below.]

The North American and South Asian samples differed in numerous ways (e.g., age, religiosity). The two groups also differed, as expected, in self-reported collectivism: (*M*_South Asians_ = 6.33) vs. (*M*_North Americans_ = 5.46), *t*(283) = 5.25, *p* < .001, η_p_^2^ = .09. To what extent was the relationship between culture and judgment of responsibility statistically mediated by collectivism? To investigate this question, we conducted a bootstrapping mediation analysis with 5000 resamples in each condition with culture (North American vs. South Asian) as the predictor, the moral sanction index as the dependent variable, and collectivism (and subsequently, individualism) as the mediator [37]. All confidence intervals were bias-corrected and accelerated (BCa).

We first focused on the Distal Intent Greater condition (in which North Americans gave higher responsibility ratings than South Asians). The mediational model examined (a) the effect of culture on moral judgment, (b) the effect of culture on collectivism, and (c) the effect of collectivism on moral judgment, controlling for culture. The confidence interval for the indirect effect, [-0.15, -0.97] did not include 0, indicating that the difference between North Americans and South Asians in collectivism contributed significantly to their difference in judgment, *p* = .0014. (For details on the computation of partial posterior *p* values in mediational models, see [38]). These results indicate for participants in the Distal Intent Greater condition, the higher the collectivism, the more lenient the judgment.

North Americans (*M* = 7.15) reported higher horizontal individualism than did South Asians (*M* = 6.65) *t*(286) = 2.33, *p* = .02, η_p_^2^ = .02. However, South Asians (*M* = 6.45) reported significantly higher vertical individualism than did North Americans (*M* = 4.92), *t*(286) = 7.43, *p* < .001, η_p_^2^ = .16, reflecting a higher sensitivity to one’s position in the status hierarchy. Mediational analyses analogous to the one above indicated that neither horizontal individualism nor vertical individualism mediated the effect of culture on judgment in any of the scenario conditions (all confidence intervals for the indirect effect included zero). Such data suggest that individualism, a complex, multiply-determined construct, does not shape people’s sensitivity to proximal and distal intent. We encourage further research into the question of why collectivism, but not individualism, appears to play a more prominent role in these effects.

In summary, participants’ sensitivity to the presence/absence of distal intent in the actor’s mind was mediated by participants’ degree of self-identified collectivism. Note that evidence of statistical mediation is not meant to imply causality (i.e., that collectivism causes less emphasis on distal intent). Instead, these data suggest that, while North Americans and South Asians differ in numerous ways, the observed between-group differences in judgments of responsibility covaried meaningfully with between-group differences in self-rated collectivism.

## Study 3

As noted, researchers have found certain commonalities between, on the one hand, males and individualistic cultures and, on the other hand, females and collectivist cultures [28–29]. While acknowledging the myriad differences between, for example, the group “East Asians” and the group “females,” we examined whether males would place more emphasis on distal intent and females more emphasis on proximal intent. If so, and if males and females in the sample did not differ in ethnic makeup, this would provide converging support for the idea that *culture*–as opposed to *ethnicity*–is behind the observed effects.

### Participants

Three hundred and ninety-two American participants (191 males) were recruited via Mechanical Turk in exchange for $0.50. Sample size was determined by calculating the amount of participants needed to achieve a minimum of 80% power based on effect sizes reported in Studies 1–2. For demographic information (including ethnicity), see Table 1. Performing the same pre-processing procedures as in Study 2 led to the removal of two participants before any data analyses were conducted. Analyses that include these participants yield results that do not differ meaningfully from the results reported below.

### Procedure

First, participants completed a short demographics form that included self-identified sex/gender. Next, for further generalizability, we used a third set of scenarios. In these scenarios, participants read about “J.G” who wished to kill his uncle to gain his inheritance (see [9]). In the “Both Present” scenario, J.G. consciously executed his planned method of killing his uncle (running him over with his car) with full knowledge that doing so would result in his uncle’s death. In the “Distal Intent Greater” scenario, J.G. had the goal of killing his uncle in mind at the exact moment that he killed his uncle without awareness that he was doing so. In the “Proximal Intent Greater” scenario, J.G. performed what turned out to be the death blow with awareness and control (i.e., pressing the accelerator), but he was not at the time thinking about killing his uncle (i.e. his mind was on his favorite song). In the “Both Absent” scenario, he neither performed the deathblow with awareness and control (he accidently ran over a pedestrian) nor had the malevolent goal in mind (his mind was on his favorite song). To summarize, the proximal intent manipulation was: pressing the accelerator on purpose versus pressing the accelerator by accident. The distal intent manipulation was: thinking about killing his uncle versus thinking about his favorite song. (Note that even though J.G.’s longstanding desire to kill his uncle was stated at the beginning of every scenario, in the conditions when Distal Intent was minimized, the intention to kill his uncle and gain the inheritance was less prominent in conscious awareness.) See S1 Appendix.

After reading the scenario, participants indicated their rating of the actor (on 0–6 scales) on the moral judgment items used in Study 1. In addition, we included six new items to provide a more nuanced measure of distinct facets of moral judgment. These included items assessing punishment (“To what extent should J.G. be punished for his action?”), justice (“To what extent is J.G. likely to get the punishment he deserves?” “To what extent does J.G. now have ‘bad karma’ as a result of what occurred?”), wrongness (“To what extent was what J.G. did fundamentally wrong?”), the actor’s view of the outcome (“How pleased is J.G. about what happened?”), and the actor’s moral character (“To what extent is J.G. a bad person?”).

### Results

#### Overall Effects

The items on the four-item moral judgment index were highly correlated (α = .74). Participants’ scores on this index were submitted to a 2 (gender: male vs. female) x 2 (distal intent: high vs. low) x 2 (proximal intent: high vs. low) between-subjects ANOVA. This revealed main effects for proximal intent, *F*(1, 390) = 16.18, *p* < .001, η_p_^2^ = .04, and distal intent, *F*(1, 390) = 281.66, *p* < .001, η_p_^2^ = .42. As in the previous studies, participants rated the actor with both forms of intent (*M* = 5.87) more responsible than the actor with neither form of intent (*M* = 3.42), *F*(1, 390) = 225.10, *p* < .001, η_p_^2^ = .56, the actor with proximal intent greater (*M* = 4.10), *F*(1, 390) = 112.71, *p* < .001, η_p_^2^ = .40, and (marginally) the actor with distal intent greater (*M* = 5.58), *F*(1, 390) = 12.90, *p* = .09, η_p_^2^ = .04.

Analyses using the 10-item moral judgment index yielded results that closely paralleled those with the 4-item index [e.g., main effects for distal intent, *F*(1, 390) = 215.11, *p* < .001, η_p_^2^ = .39 and proximal intent, *F*(1, 390) = 8.85 *p* < .001, η_p_^2^ = .07, distal intent x gender interaction, *F*(1, 390) = 2.84, *p* < .09, η_p_^2^ = .01.] Given the largely parallel results, we report the 4-item for the sake of consistency among all four studies. A complete table of participants’ ratings for each of the ten items is presented in S2 Appendix.

#### Effects Involving Gender

There was not a significant main effect of gender, *F*(1, 390) = 0.88, indicating that males and females did not differ overall in how harshly they judged the actor. However, a significant distal intent x gender interaction, *F*(1, 390) = 4.58, *p* = .03, η_p_^2^ = .01, indicated that males’ and females’ judgments differed depending on the degree to which distal intent was present versus absent.

Analogously to Studies 1 and 2, we probed this interaction by comparing males and females across the two scenarios in which one form of intent was more prominently in mind than the other form of intent. A 2 (scenario condition: Distal Intent Greater versus Proximal Intent Greater) x 2 (gender: male versus female) ANOVA revealed a significant interaction, *F*(1, 186) = 4.02, *p* < .05, η_p_^2^ = .02. As depicted in Table 4, the pattern was in the predicted direction: Males judged the actor more harshly when distal intent was present and proximal intent was absent whereas females judged him more harshly when proximal intent was present and distal intent was absent.

**Table 4:** Moral sanction of the actor. (Standard deviations in parentheses.)

	Female			Male	
	Distal Intent			Distal Intent	
Prox. Intent	High	Low	Prox. Intent	High	Low
High	5.91 (0.40)	4.24 (1.41)	High	5.82 (0.46)	3.89 (1.61)
Low	5.38 (1.24)	3.60 (1.55)	Low	5.76 (0.43)	3.22 (1.39)

Although there are innumerable ways in which the groups “men” and “Western cultures” differ and “women” and “Eastern cultures” differ, these data are consistent with previous findings indicating that, in the aggregate, men respond in a slightly more “Western” fashion than women do (e.g., [28–29]. Here, when judging an actor, men placed greater weight on the actor’s distal intent, just as North Americans did in Study 1 and Study 2, whereas women placed greater weight on proximal intent, just as East Asians did in Study 1 and South Asians did in Study 2. Given the results of Study 3, the fact that in Study 1 the Chinese sample was disproportionately male and the Canadian sample disproportionately female should have militated against obtaining differences between the Chinese and Canadian groups. That we observed the Chinese vs. Canadian difference despite an unfavorable gender breakdown strengthens our confidence in the cross-cultural effect.

In summary, by operationalizing “culture” via gender, Study 3 provides an additional form of evidence that that some cultures tend to prioritize the actor’s proximal intent in moral judgment, whereas other cultures tend to prioritize distal intent.

## Study 4

In Study 4 we examined whether the gender difference found in Study 3 would extend to positive acts. Do perceivers consider proximal and distal intent when determining how much praise to give actors who perform meritorious acts? How do people judge an actor who had a positive distal intent in mind but achieved the outcome via serendipitous means? How do people rate an actor who consciously performed an act that turned out to be serendipitously positive? Are there asymmetries in the judgment of negative versus positive actions (e.g., [39]) or do the gender differences observed in Study 3 affect perceptions of proximal and distal intent for positive acts in the same manner as they do for negative acts?

In addition, it is likely that people make intentionality judgments in mundane, morally neutral situations. In this study we examined whether perceivers would apply the PIDI framework to understand morally neutral acts. If so, this would suggest that the PIDI framework represents a general model of intent perception, not restricted to moral acts. If not, it would suggest that people only apply the PIDI model when they make judgments about morally unacceptable versus acceptable behavior. To examine this question, we created scenarios in which the actor scored a soccer goal with both proximal and distal intent in mind, with proximal intent greater than distal intent, with distal intent greater than proximal intent, or with both forms of intent absent.

### Participants

Four hundred and sixty-eight American participants (206 males) were recruited via Amazon Mechanical Turk in exchange for a payment of US $0.50 (mean age = 30.55). Sample size was determined by using the sample size and effect sizes of Study 3 as reference points. We decided to add approximately 80 more participants given that, in Study 3, within-condition simple effects tests of gender did not reach significance. For more complete demographic information (including ethnicity), see Table 1. Pre-processing of the raw data using the procedures used in Studies 2–3, led to the elimination of three participants prior to statistical analysis. Analyses that include these participants yielded results that did not differ meaningfully from the results reported below.

### Procedure

Participants were randomly assigned to read one of four scenarios. At the start of each scenario, the text indicated that “Jane’s soccer team is tied with its arch-rival with one minute left in the championship game. Jane desperately wants her team to score a goal and win the championship. Jane has the ball at her feet near the opponent’s goal.” In the Both Present scenario, Jane aims for the corner of the goal and with precision and control kicks the ball into the net. In the Distal Intent Greater condition, she aims for the corner of the goal but the ball veers off course, bounces off a defender, and goes into the net for a goal. In the Proximal Intent Greater condition, she aims to pass the ball to her teammate standing in front of the goal, accurately kicks the ball toward her teammate, but neither the teammate nor the goalkeeper are expecting the pass, so the ball goes past them both into the net for a goal. In the Both Absent condition, Jane aims to pass the ball to her teammate, but the ball veers off course, bounces off a defender, and goes into the net for a goal. In summary, the proximal intent manipulation was whether Jane kicked the ball toward her target in a controlled, accurate manner versus an uncontrolled, inaccurate manner. The distal intent manipulation was whether Jane was aiming for the goal versus aiming for her teammate. (For the full text, see S1 Appendix)

Next, participants rated Jane on (0–6 scales) on items used in Study 1 that were re-worded for a positive outcome: (1) “How intentional was Jane’s action?” (2) “How responsible was Jane for the goal?” (3) “How positively should Jane be viewed?” and (4) “How much praise does Jane deserve?” (0–6 scales).

Finally, participants completed a brief demographics form that included sex/gender.

### Results

#### Overall Effects

Participants’ scores on the judgment index (α = .70) were submitted to a 2 (gender: male vs. female) x 2 (distal intent: high vs. low) x 2 (proximal intent: high vs. low) between-subjects ANOVA. This revealed main effects for proximal intent, *F*(1, 462) = 128.20, *p* < .001, η_p_^2^ = .22, and distal intent, *F*(1, 462) = 67.32, *p* < .001, η_p_^2^ = .13. Following the pattern of Studies 1–3, participants rated the actor with both forms of intent in mind (M = 5.15) more responsible than the actor with neither form of intent in mind (M = 3.61), *F*(1, 462) = 176.84, *p* < .001, η_p_^2^ = .48, the actor with distal intent more prominent (M = 3.79), *F*(1, 462) = 147.40, *p* < .001, η_p_^2^ = .44, and the actor with proximal intent more prominent (M = 4.07), *F*(1, 462) = 100.35, *p* < .001, η_p_^2^ = .36.

#### Effects Involving Gender

There was not a significant main effect of gender, *F*(1, 462) = 0.90, indicating that males and females did not differ overall in how favorably they judged the actor. However, the analysis revealed a significant proximal intent x gender interaction, *F*(1, 462) = 4.80, *p* < .03, η_p_^2^ = .01, and a marginally significant distal intent x gender interaction, *F*(1, 462) = 3.25, *p* = .07, η_p_^2^ = .007.

Analogously to Studies 1–3, we compared males and females across the two scenarios when only one form of intent was present. A 2 (scenario condition: Distal Intent Greater vs. Proximal Intent Greater) x 2 (gender: male versus female) ANOVA found a significant effect, *F*(1, 254) = 8.37, *p* < .01, η_p_^2^ = .03, indicating that males and females differed reliably as to which one type of intent (proximal or distal) mattered most.

Simple effects tests revealed that the pattern fell in the predicted direction. When only proximal intent was in Jane’s mind, females (*M* = 4.22) judged her more favorably than did males (*M* = 3.88), *F*(1, 462) = 5.71, *p* < .02, η_p_^2^ = .04. In contrast, when only distal intent was in her mind, males (*M* = 3.95) judged her marginally more favorably than did females (*M* = 3.66) *F*(1, 462) = 3.36, *p* < .07, η_p_^2^ = .03. Males’ and females’ judgments did not differ when Jane had neither form of intent in mind, *F*(1, 462) = .30, or both forms of intent in mind, *F*(1, 460 = .84). See Table 5.

**Table 5:** Praise of the actor. (Standard deviations in parentheses.)

	Female			Male	
	Distal Intent			Distal Intent	
Prox. Intent	High	Low	Prox. Intent	High	Low
High	5.22 (0.88)	4.22 (0.85)	High	5.07 (0.65)	3.88 (0.78)
Low	3.66 (0.88)	3.65 (1.03)	Low	3.95 (0.91)	3.56 (0.96)

Given the gender effects reported in Studies 3 and 4, readers might wonder whether a reanalysis of the Study 1 and Study 2 data that included gender might yield further supporting evidence. However, in Study 1 both the Canadian and Chinese student samples were so heavily skewed (Canadians = 72% female; Chinese = 78% male) that meaningful analyses involving gender could not be performed. Similarly, in Study 2 the South Asian sample was 68% male, yielding too few females for a valid comparison. The most evenly-distributed samples in terms of gender were the North American MTurk samples used in Studies 3 and 4.

In summary, males in Study 4 followed the same pattern displayed by North Americans in Studies 1–2 and males in Study 3: greater sensitivity to the presence or absence of distal intent. Females followed the same pattern displayed by East Asians (Study 1), South Asians (Study 2), and females (Study 3): greater sensitivity to the presence or absence of proximal intent. The fact that this pattern held even when the actor performed a positive, morally-neutral act (scoring a soccer goal) suggests that the PIDI framework may not be restricted to moral judgment. Instead, it may characterize how laypeople determine intentionality more generally. It is important to note, however, that the scenarios in Study 4 differed from the scenarios in Studies 1–3 in other aspects as well, including severity. Whereas in Studies 1–3, the scenarios involved death, the scenario in Study 4 was both more innocuous and positive. Future researchers may consider empirically teasing apart valence and severity.

## General Discussion

In Study 1, as long as the actor had the malevolent goal prominently in mind at the time of the act, Canadians judged her morally responsible even if the outcome transpired by accident. For the Chinese participants, as long as the actor performed the action with awareness and control, they judged her morally responsible even if she was not at that moment thinking about her malevolent goal. In Study 2, we found a similar pattern when we replaced Chinese participants with South Asian participants. In Study 3, as long as the actor had the malevolent goal prominently in mind, males judged him more responsible than females, even if the outcome transpired by accident. In Study 4, this pattern extended to a positive, morally-neutral act: as long as the actor was thinking about scoring a goal, males judged her more favorably than did females, even if the outcome transpired by accident. In contrast, as long as the actor performed the movement with awareness and control, females judged her more favorably than did females, even if the actor was not thinking about scoring a goal at that moment.

These data suggest that the concepts of proximal and distal intent are intuitive to most people. The inclusion or exclusion of these ingredients significantly influenced judgments of responsibility and condemnation/praise. Thus, these data represent evidence that proximal and distal intent are core ingredients of lay reasoning about intentionality and responsibility. The data go on to suggest that while the ‘raw materials’ of moral reasoning may be largely uniform across culture [40], different cultures may encourage differential emphasis on specific elements.

### Limitations and Future Directions

In these studies, we found significant cultural differences in judgments of responsibility when culture was operationalized in the broadest of brushstrokes (i.e. continent, gender). Indeed, most studies in the history of cultural psychology have used some form of an “East vs. West” dimension as their primary independent variable. However, while broad East versus West ‘main effects’ have proven useful, a number of recent researchers have accounted for more variability by considering nuanced interactions between nationality and other ‘generic’ situational, motivational, and individual difference variables (e.g., [41–43]). To cite one example, Leung and D. Cohen [44] demonstrated that the same personality trait may manifest itself in diametrically opposing ways in different cultures.

One variable that likely interacts with broad variables like nationality is religious tradition. A. Cohen and colleagues, for example, have highlighted the distinction between “thought-focused” and “action-focused” religious traditions that may exist within the same nation, such as the U.S. [19, 5]. In support of this idea, A. Cohen and Rozin [19] found that while American Protestants and American Jews rated a person who intentionally committed an immoral act equally negatively, the Protestants rated a person who merely had immoral thoughts (but did not act on those thoughts) more negatively than did the Jews. Put in the present terms, Protestants may place more emphasis than Jews on distal intent. In ongoing studies, we are investigating the interplay of culture *and* religion on judgments of intent. Such an investigation may yield important differences between, say, participants in China (where religious identification is low) and participants in India (where religious identification is high).

In addition, a number of researchers have questioned the assumed dispositional fixedness and generalizability of conventional cultural dichotomies such as collectivism/individualism and independence/interdependence. For example, Oyserman et al.’s [45] ‘situated cognition’ approach argues that many cultural differences are traceable to mindsets that are activated by momentary cues. (See also [46].) Although in Study 2 we found that the cross-cultural effect was significantly related to differences in self-reported collectivism, we do not wish to imply that collectivism is necessarily a fixed trait. Consistent with social-cognitive approaches to knowledge activation (e.g., [47]), we suggest that cultural mindsets are susceptible to both ‘chronic’ and ‘temporary’ forms of activation. As such, individuals’ prepotent, culturally-shaped processing tendencies may be superseded—at least temporarily—by compelling cues in the environment. In future studies, researchers should consider examining whether the effects reported here replicate with situationally-induced cultural primes.

Moreover, while in the present studies culture was the independent variable and judgment of responsibility was the dependent variable, there are good reasons to believe that the reverse association may also apply. That is, the act of making particular types of moral judgments may serve to shape, encourage, and strengthen particular cultural norms and worldviews. Future researchers would do well to examine this possibility. Might it be the case, for example, that encouraging bicultural participants (e.g., ethnic Chinese in the United States) to focus on distal intent will cause them to identify more strongly as American while encouraging a focus on proximal intent will cause them to identify more strongly as Chinese?

## Conclusion

The PIDI framework is generative in that it may used to test a range of future hypotheses. For example, how (if at all) might the pattern vary depending on whether the actor is an ingroup versus outgroup member? How (if at all) might the pattern vary depending on whether the *victim* is an ingroup versus outgroup member? How (if at all) might the pattern vary depending on whether the transgression is a “purity violation” versus other types of norm violations [48]? How might the pattern vary depending on participants’ age [49]? These are topics of current research in our laboratory.

## Supporting Information

S1 Appendix (DOCX)

S2 Appendix (DOCX)
