# Supplementary material for: Effects of Culture and Gender on Judgments of Intent and Responsibility
Source: PLoS One. 2016 Apr 28;11(4):e0154467. doi: 10.1371/journal.pone.0154467 (PMC4849663; doi:10.1371/journal.pone.0154467)
Supplement: S1 Appendix — (DOCX) [file pone.0154467.s001.docx]

**APPENDIX A**

Scenarios used in Study 1.

**Both Present**

Barbara wants to kill her husband, John. She formulates a plan to poison him while dining at a local restaurant. At one point during the meal, Barbara, thinking about killing John, slips some poison from the vial in her purse into John’s dish while he is away at the restroom. John returns and takes a bite of the now-poisoned food. The poison kills him virtually instantly.

**Distal Intent Greater**

Barbara wants to kill her husband, John. She formulates a plan to poison him while dining at a local restaurant. At one point during the meal, Barbara, thinking about killing John, slips some poison from the vial in her purse into John’s dish while he is away at the restroom. John returns and takes a bite of the now-poisoned food. The poison is not strong enough to kill John. Instead, it makes the dish taste so bad that John changes his order. His

new dish contains a food that he is extremely allergic to. John eats the new dish and it kills him virtually instantly.

**Proximal Intent Greater**

Barbara wants to kill her husband, John. She formulates a plan to poison him while dining at a local restaurant. At one point during the meal, John goes away to the restroom. Suddenly a spider crawls across John’s plate. Barbara hates spiders. Barbara, thinking only about killing the spider, takes the poison vial from her purse and drowns the spider on John’s plate. John returns and takes a bite of the now-poisoned food. The poison kills him virtually instantly.

**Both Absent**

Barbara wants to kill her husband, John. She formulates a plan to poison him while dining at a local restaurant. At one point during the meal, John goes away to the restroom. Suddenly a spider crawls across John’s plate. Barbara hates spiders. Barbara, thinking only about killing the spider, takes the poison vial from her purse and drowns the spider on John’s plate. John returns and takes a bite of the now-poisoned food. The poison is not strong enough to kill John. Instead, it makes the dish taste so bad that John changes his order. His new dish contains a food that he is extremely allergic to. John eats the new dish and it kills him virtually instantly.

Scenarios used in Study 2.

**Both Present**

Alex wants to kill his ex-girlfriend Linda. He formulates a plan to drown her in the lake during a camping trip they attend with the same group of friends every year. Knowing she can swim, Alex plans to tie her up with a rope before pushing her into the water. During their trip Alex convinces Linda to go for a boat ride with him. As they are about to get into the boat, docked in a secluded area by the lake, Alex takes a rope from the boat, ties Linda up and pushes her into the lake. Linda drowns.

**Distal Intent Greater**

Alex wants to kill his ex-girlfriend Linda. He formulates a plan to drown her in the lake during a camping trip they attend with the same group of friends every year. Knowing she can swim, Alex plans to tie her up with a rope before pushing her into the water. During their trip Alex convinces Linda to go for a boat ride with him. As they are about to get into the boat, docked in a secluded area by the lake, Alex takes a rope from the boat, and begins to tie her up. In trying to free herself, Linda manages to push Alex into the water and start running away. However, she trips on the dock, hits her head, falls unconscious into the water and drowns.

**Proximal Intent Greater**

Alex wants to kill his ex-girlfriend Linda. He formulates a plan to drown her in the lake during a camping trip they attend with the same group of Linda every year. During their trip Alex convinces Linda to go for a boat ride with him. As they are about to get into the boat, Alex notices the safety rope is tangled. Thinking that he will later need the rope, he pulls it out so that they can untangle it. After thinking of the best method of untangling the rope, he directs Linda to hold onto one end and tries to untangle the rest by temporarily wrapping the rope around her. Unbeknownst to both of them, when Alex pulls on the rope it becomes wrapped a little too tight around Linda. It cuts off blood circulation so that she very quickly loses consciousness, falls into the lake and drowns.

**Both Absent**

Alex wants to kill his ex-girlfriend Linda. He formulates a plan to drown her in the lake during a camping trip they attend with the same group of friends every year. During their trip Alex convinces Linda to go for a boat ride with him. As they are about to get into the boat, Alex notices the safety rope is tangled. He pulls it out so that they can untangle it. As they are untangling the rope, Linda trips, gets tangled in the rope, falls into the lake and drowns.

Scenarios used in Study 3.

**Both Present**

J.G. wished to kill his rich uncle, as he stood to inherit a large sum of money. He formulated a plan to kill uncle at his uncle’s home by running him down with his car. He began to drive speedily to his uncle’s home. As he drove, he thought about killing his uncle. J.G. arrived at the house, saw his uncle in front, and intentionally pressed the accelerator. The car struck his uncle, killing him instantly.

**Distal Intent Greater**

J.G. wished to kill his rich uncle, as he stood to inherit a large sum of money. He formulated a plan to kill uncle at his uncle’s home by running him down with his car. He began to drive speedily to his uncle’s home. As he drove, he thought about killing his uncle. As he approached his uncle’s house, J.G. noticed a person crossing into the path of the car. Startled to see a person in the road, J.G. tried to press the brake but pressed the accelerator instead. The car struck the pedestrian, killing him instantly. The pedestrian turned out to be J.G.’s uncle.

**Proximal Intent Greater**

J.G. wished to kill his rich uncle, as he stood to inherit a large sum of money. He formulated a plan to kill uncle at his uncle’s home by running him down with his car. He began to drive speedily to his uncle’s home. A he drove, he calmed his nerves by focusing on his favorite song. As he approached his uncle’s house, J.G. noticed a person crossing into the path of the car. J.G. tried to drive past the person in the road by intentionally pressing the accelerator. The car struck the pedestrian, killing the pedestrian instantly. The pedestrian turned out to be J.G.’s uncle.

**Both Absent**

J.G. wished to kill his rich uncle, as he stood to inherit a large sum of money. He formulated a plan to kill uncle at his uncle’s home by running him down with his car. He began to drive speedily to his uncle’s home. A he drove, he calmed his nerves by focusing on his favorite song. As he approached his uncle’s house, J.G. noticed a person crossing into the path of the car. Startled to see a person in the road, J.G. tried to press the brake but pressed the accelerator instead. The car struck the pedestrian, killing him instantly. The pedestrian turned out to be J.G.’s uncle.

Scenarios used in Study 4.

**All Scenarios**

Jane’s soccer team is tied with its arch-rival with one minute left in the championship game. Jane desperately wants her team to score a goal and win the championship. Jane has the ball at her feet near the opponent’s goal.

**Both Present**

Spotting an opening, Jane aims to shoot the ball toward the corner of the net. With precision and control, she kicks the ball right past the goalkeeper and into the corner of the net for a goal. Her team wins the game and the championship.

**Distal Intent Greater**

Spotting an opening, Jane aims to shoot the ball toward the corner of the net. She kicks the ball, but it veers off course. However, it bounces off the leg of a defender and goes right past the goalkeeper and into the corner of the net for a goal. Her team wins the game and the championship.

**Proximal Intent Greater**

Spotting an opening, Jane aims to pass the ball to a teammate who is standing between her and the goal. With precision and control, she kicks the ball right to her teammate. Her teammate is not expecting the pass and doesn’t see the ball zoom by her. However, the teammate is also blocking the goalkeeper’s view. The ball continues right past the goalkeeper and into the corner of the net for a goal. Her team wins the game and the championship.

**Both Absent**

Spotting an opening, Jane aims to pass the ball to a teammate who is standing between her and the goal. She kicks the ball, but it veers off course. However, it bounces off the leg of a defender and goes right past the goalkeeper into the corner of the net for a goal. Her team wins the championship and the game.
