# Supplementary material for: Effects of Culture and Gender on Judgments of Intent and Responsibility
Source: PLoS One. 2016 Apr 28;11(4):e0154467. doi: 10.1371/journal.pone.0154467 (PMC4849663; doi:10.1371/journal.pone.0154467)
Supplement: S2 Appendix — (DOCX) [file pone.0154467.s002.docx]

**APPENDIX B**

*Study 3.* Moral sanction of the actor as a function of gender and the relative presence or absence of proximal intent and distal intent – *all ten dependent measure items* (standard deviations in parentheses)

| Sex | DI | PI | To what extent were Alex’s actions intentional? | How much responsibility does Alex deserve? | How negatively should Alex be judged? | How much blame should Alex receive? | To what extent does Alex now have ‘bad karma’? | How likely is it that Alex will receive punishment | How pleased is Alex about what happened? |
| --- | --- | --- | --- | --- | --- | --- | --- | --- | --- |
| Female | low | Low | 3.16 (1.75) | 3.76 (1.69) | 3.84 (1.67) | 3.65 (1.71) | 3.80 (1.96) | 3.43 (1.79) | 5.05 (1.41) |
|  |  | High | 3.30 (1.65) | 4.55 (1.57) | 4.52 (1.53) | 4.56 (1.89) | 3.86 (1.76) | 4.36 (1.52) | 4.98 (1.34) |
|  | high | Low | 5.51 (1.20) | 5.27 (1.47) | 5.42 (1.27) | 5.34 (1.33) | 4.73 (1.86) | 5.44 (0.97) | 4.94 (1.45) |
|  |  | High | 5.93 (0.32) | 5.92 (0.46) | 5.87 (0.54) | 5.93 (0.32) | 4.23 (2.08) | 5.60 (0.92) | 4.83 (1.29) |
| Male | low | Low | 2.76 (1.57) | 3.25 (1.54) | 3.51 (1.68) | 3.33 (1.77) | 3.37 (1.92) | 3.02 (1.83) | 5.02 (1.19) |
|  |  | High | 3.08 (1.74) | 4.14 (1.90) | 4.27 (1.84) | 4.14 (1.89) | 3.70 (2.01) | 4.05 (1.88) | 4.57 (1.59) |
|  | high | Low | 5.76 (0.56) | 5.79 (0.53) | 5.63 (0.77) | 5.84 (0.41) | 3.79 (2.16) | 5.23 (1.13) | 4.73 (1.60) |
|  |  | High | 5.89 (0.38) | 5.89 (0.43) | 5.63 (0.89) | 5.89 (0.42) | 4.02 (2.20) | 5.42 (1.13) | 4.47 (1.67) |
| Sex | DI | PI | To what extent did Alex do it “on purpose”? | Will Alex get what he deserves? | Should Alex receive punishment? |  |  |  |  |
| Female | low | Low | 3.29 (1.66) | 3.48 (1.73) | 3.65 (1.76) |  |  |  |  |
|  |  | High | 3.79 (1.59) | 3.31 (1.74) | 4.45 (1.69) |  |  |  |  |
|  | high | Low | 5.62 (0.91) | 4.11 (1.81) | 5.67 (0.97) |  |  |  |  |
|  |  | High | 5.94 (0.32) | 3.98 (1.98) | 5.79 (0.66) |  |  |  |  |
| Male | low | Low | 3.33 (1.77) | 3.33 (1.76) | 3.35 (1.70) |  |  |  |  |
|  |  | High | 4.14 (1.89) | 3.11 (1.95) | 4.30 (1.92) |  |  |  |  |
|  | high | Low | 5.84 (0.41) | 3.11 (1.82) | 5.75 (0.63) |  |  |  |  |
|  | high | High | 5.89 (0.42) | 3.38 (1.94) | 5.87 (0.56) |  |  |  |  |
